# Infrared spectrum analysis of organic molecules with neural networks using standard reference data sets in combination with real-world data

**DOI:** 10.1186/s13321-025-00960-2

**Published:** 2025-02-26

**Authors:** Dev Punjabi, Yu-Chieh Huang, Laura Holzhauer, Pierre Tremouilhac, Pascal Friederich, Nicole Jung, Stefan Bräse

**Affiliations:** 1 Institute of Biological and Chemical Systems, Karlsruhe Institute of Technology (KIT), Kaiserstraße 12, 76131 Karlsruhe, Germany; 2 Institute of Theoretical Informatics, Karlsruhe Institute of Technology (KIT), Kaiserstraße 12, 76131 Karlsruhe, Germany; 3 Institute of Nanotechnology, Karlsruhe Institute of Technology (KIT), Kaiserstraße 12, 76131 Karlsruhe, Germany; 4 Karlsruhe Nano Micro Facility (KNMFi), Karlsruhe Institute of Technology (KIT), Kaiserstraße 12, 76131 Karlsruhe, Germany; 5 Institute of Organic Chemistry, Karlsruhe Institute of Technology (KIT), Kaiserstraße 12, 76131 Karlsruhe, Germany

**Keywords:** Infrared spectra, Machine learning, Data analysis, Open databases

## Abstract

In this study, we propose a neural network- based approach to analyze IR spectra and detect the presence of functional groups. Our neural network architecture is based on the concept of learning split representations. We demonstrate that our method achieves favorable validation performance using the NIST dataset. Furthermore, by incorporating additional data from the open-access research data repository Chemotion, we show that our model improves the classification performance for nitriles and amides.

**Scientific contribution**: Our method exclusively uses IR data as input for a neural network, making its performance, unlike other well-performing models, independent of additional data types obtained from analytical measurements. Furthermore, our proposed method leverages a deep learning model that outperforms previous approaches, achieving F1 scores above 0.7 to identify 17 functional groups. By incorporating real-world data from various laboratories, we demonstrate how open-access, specialized research data repositories can serve as yet unexplored, valuable benchmark datasets for future machine learning research.

**Supplementary Information:**

The online version contains supplementary material available at 10.1186/s13321-025-00960-2.

## Introduction

In molecular chemistry, functional groups are substructures consisting of a few atoms with a characteristic composition and structure. Within the molecule, functional groups are often a reactive part and define or at least influence the chemical properties of compounds. Determining the presence or absence of functional groups is important for chemists to confirm the structure of a synthesized compound. The measurement of infrared (IR) spectra is one of the most established methods to identify functional groups within organic molecules, even in those cases where the most common techniques such as NMR spectroscopy fail. Infrared (IR) spectra are obtained as a result of the interaction of infrared light with molecules. The absorption of infrared radiation induces molecular vibrations, i.e. periodic distortions of the molecular structure, including stretching, contracting, bending, and torsional motions of bonds, angles, dihedral angles, and other collective variables. By analyzing spectral parameters such as the position of the absorption energy band, the bandwidth, and the absorption coefficient, valuable information about the structure and functional groups of the molecule can be deduced [11, 26, 27].

The classical analysis of IR spectra [5, 10, 28] is based on metrics such as position, intensity, area, and width of peaks to describe absorption bands and gather information about the molecular concentration and bonding environment. Interpreting an infrared spectrum requires expert knowledge to correlate the spectrum with the substructures of the molecules and to account for the relative environment and molecular structure. However, this knowledge-based manual approach can be challenging to formalize and implement, specifically when aiming to apply it to complex systems with overlapping absorption signals from different chemical species. Therefore, the classical (manual) analysis of IR data is resource-intensive and time-consuming, allowing only a low throughput of analytical investigations. These limitations hamper the interpretation of complex IR spectra and the systematic and fast interpretation needed for high-throughput analysis of samples by IR spectroscopy. Recently, computational methods have been playing an increasing role in analyzing IR spectra, bringing together advancements in machine learning and quantum chemistry. These methods help in accurately predicting and interpreting intricate IR spectra, making it easier to identify molecular structures. Traditional computational methods (e.g. [12]) include e.g. the option to implement efficient forward spectra prediction models and the potential to integrate with extensive databases. Key advantages of using computational methods compared to manual analysis are the increase in speed, the increase in accuracy and reproducibility. Nevertheless, also traditional computational techniques have their limitations which are e.g. the dependency of handcrafted descriptors based on expert knowledge to extract relevant spectral signals.

Machine learning could solve the limitations of the more traditional methods to calculate and analyze spectroscopic data as it offers to learn descriptors directly from the available data. The versatility and potential of ML in advancing chemical research was demonstrated by many examples in the past, e.g. by the application of machine learning for molecular property prediction and molecular design [1, 13, 17, 18, 29, 31]. One of the earliest applications of artificial neural networks to spectral analysis was to find functional groups from IR spectra, where the authors Fessenden and Györgyi [8] used a 2-layer feed-forward neural network. The field of machine learning has come a long way since then, with new optimization algorithms and model architectures, delivering state-of-the-art performance. Other attempts [e.g. 2] implemented similarity search algorithms that harnessed machine learning techniques to extract feature vectors for comparison. Wang et al. [30] used support vector machines to improve the prediction accuracy on a database exported from the software Omnic by Thermofisher Scientific [22]. Enders et al. [7] published a method which uses one convolutional neural network per functional group type to find functional groups present in the IR spectra. Their work uses spectra images as input, and therefore can potentially suffer from problems such as activation due to non-spectra pixels in the image and loss of information due to max pooling. Fine et al. [9] combined mass spectra data and IR data at the input of their models. Their model consists of a combination of an auto-encoder [32] and a densely connected neural network. The auto-encoder learns embeddings as a function of mass spectra and IR spectra, which are then further processed by following fully connected layers to produce predictions.

Currently, the datasets available for machine learning on IR spectroscopic data are limited. Following, we describe the largest and most known databases that are available for IR spectroscopy:

- NIST SRD catalog [21] : NIST (National Institute of Standards and Technology, US Department of Commerce) produces the Nation’s Standard Reference Data (SRD). NIST provides 49 free SRD databases and 41 fee-based SRD databases (status as of year 2024). All these databases can be viewed under the catalog NIST SRD catalog [21]. NIST SRD 69 [20] is an online library that provides access to a diverse range of spectroscopic data, including IR spectra, in the form of downloadable files in jcamp-dx format which have to be purchased. It provides IR spectra for over 16,000 compounds. IR spectra data can be searched for specific compounds in the Chemistry WebBook (NIST SRD 69) based on name, chemical formula, CAS registry number, molecular weight, chemical structure, or selected ion energetics and spectral properties. For our study, we used NIST SRD 35 [19] which is a commercial and predefined dataset (further referred to as ’NIST’). NIST SRD 35 data collection comprises 5,228 infrared spectra of various compounds, accompanied by chemical structures for most of them. The spectra are provided in JCAMP-DX format on a CD-ROM, while the chemical structures are in MOL-file format. The infrared data originated from two sources: the “EPA Vapor-Phase IR Library” (3,108 spectra) and NIST laboratories (2,120 spectra). All spectra are presented as normalized absorbance, and empirical formulas and CAS Registry Numbers are provided for all compounds. NIST spectra were acquired at 8 $\textrm{cm}^{-1}$ resolution using an integrated capillary GC-MS-IR instrument. The data have been standardized to 8.0 $\textrm{cm}^{-1}$ resolution for consistency. EPA spectra cover the range 450-3966 cm-1, while NIST spectra range from 550-3846 $\textrm{cm}^{-1}$.
- SDBS [24]: The SDBS (Spectral Database for Organic Compounds) is an online library for organic compounds, which offers a maximum of six different types of spectra under a directory of the compounds. The available spectra types include mass spectra (EI-MS), Fourier transform IR spectra (FT-IR), 1H nuclear magnetic resonance (NMR) spectra, ^13^C NMR spectra, Raman spectra, and electron spin resonance (ESR) spectra. All the IR spectra were measured at the National Institute of Advanced Industrial Science and Technology (AIST), Japan, using a Nicolet 170SX or a JASCO FT/IR-410. The spectral resolution for the Nicolet 170SX was 0.25 $\textrm{cm}^{-1}$, and the spectral data were stored in the database at intervals of 0.5 $\textrm{cm}^{-1}$ at 4000-2000 $\textrm{cm}^{-1}$, and of 0.25 $\textrm{cm}^{-1}$ at 2000-400 $\textrm{cm}^{-1}$. The spectral resolution and the interval were 0.5 $\textrm{cm}^{-1}$ for the JASCO FT/IR-410. The SDBS library provides spectra images only.
- Sigma-Aldrich Library [25]: The Sigma-Aldrich Library of FT-IR Spectra is a comprehensive collection of FT-IR spectra sourced from the laboratories of Sigma-Aldrich by Merck KGaA Darmstadt, Germany. Featuring over 11,000 pure compounds and over 11,250 spectroscopic records. The Sigma-Aldrich Library of FT-IR Spectra includes compound properties (molecular formula, mass, compound class) and FT-IR spectroscopic data, which has been evaluated by Wiley and third-party experts. Featured spectra were measured in the spectral range of 4000 to 400 $\textrm{cm}^{-1}$ and compound classes. Sigma Aldrich’s commercial IR library is available with a yearly subscription.

As the datasets from all three databases are not openly accessible, the referencing of machine learning results to the same data is still a challenge for the work on IR data. The issue of missing datasets was temporarily solved in previous studies [7, 9, 14] by using scrapper tools to get data from the above-mentioned online libraries. Nevertheless, generating datasets through web scraping frequently introduces challenges related to data inconsistency and ethical considerations. Additionally, if the underlying databases are commercial, disclosing the specific dataset is not possible. Consequently, comparing ML methods is challenging due to potential variations in training and test data size, quality, and distribution.

## Approach

In this work, we develop a simple-to-implement and reproducible method to identify the presence of given functional groups in IR spectra based on machine learning. We employ neural networks to automate the learning of features from the data, enabling the identification of functional groups present in given molecules. Our method overcomes the disadvantages of previous work, such as being able to predict multiple functional groups from a single model, avoiding the use of images and mass spectra data in order to get higher scores. Our goal is to develop a fully automated data-driven method that can be easily integrated into electronic lab notebooks for chemistry such as Chemotion repository [3]. As a starting point, we adopt the problem definition proposed by Fine. According to that, a neural network is trained with IR data gained from the NIST SRD 35 [19] dataset to identify the presence of 17 functional groups. While other functional groups in our dataset could have been relevant, we chose not to modify the number or selection to ensure direct comparability with the previous model, though the method should be extended to include more functional groups in the future. The methodic work in this study was prepared by the generation and merging of the desired datasets to be included. While the NIST dataset could be ordered and was used as is, our open-access dataset for easily accessible IR data needed to be prepared. As a source, we used the Chemotion repository [3], which is a publicly funded research data repository hosted at the Karlsruhe Institute of Technology (KIT) in Germany. The Chemotion repository comprises experimental data obtained from the characterization of chemical compounds, such as 1H NMR data, ^13^C NMR data, IR data, mass spectrometry data, and several other techniques. The analyzed compounds were synthesized across various chemical laboratories, primarily aiming to validate findings in scientific publications. To obtain IR spectra and the related molecular structures from the Chemotion repository, we exported parts of the database content. The obtained files were further processed as described in the methods section. An alternative way to obtain data from the Chemotion repository is to fetch the whole dataset available in Chemotion via the API and to reduce the dataset to the required spectra types and related information. In the exported dataset from the Chemotion repository, there were 4175 samples with assigned IR spectra. Most of the data came from the chemical labs at the Karlsruhe Institute of Technology, with a smaller portion contributed by other research institutes. Each sample includes an IR spectrum, molecular SMILES code, and corresponding functional group labels after data preprocessing. Following our data preprocessing pipeline (mentioned in the section below) we end up with a dataset with 1763 samples. We call this preprocessed version of the dataset “Chemotion”. Chemotion holds the distinct advantage of being open-source, and readily accessible for utilization. To facilitate easy and inexpensive research in field of machine learning for IR spectrum analysis, we published the IR dataset Chemotion [15] in an open-access repository.

**Fig. 1:**
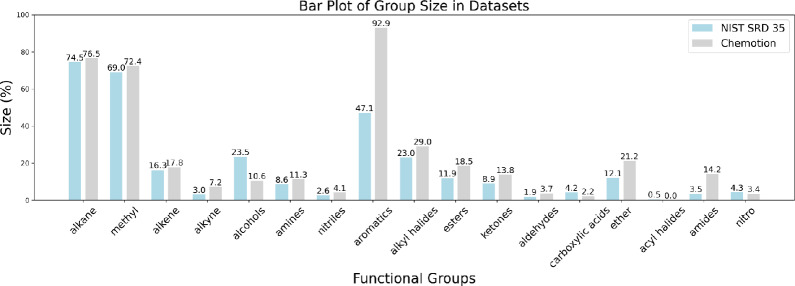
Functional group distribution in the NIST dataset. The figure shows the percentages of functional groups present in the NIST and Chemotion dataset. As there are multiple functional groups per molecule, the cumulative percentage is not 100

Much like the NIST dataset, the Chemotion dataset also exhibits class imbalance, the overall distribution of functional groups partially mirrors that of the NIST. Distribution plots provide insights into the class distribution present in the dataset which helps to analyze data-driven models. Fig. 1 provides a side-by-side comparison of the functional group distributions in the NIST and Chemotion databases. Both datasets share an imbalance, and their functional group distributions closely align. Acyl halides have the lowest representation in both the datasets with only 26 samples in NIST and Chemotion with no samples. Approximately 93% of the samples in the Chemotion dataset have aromatic groups, compared to only 47% of the samples in NIST. The Chemotion dataset contains about 10% more samples with an amide group. NIST dataset contains approximately 10% more samples with alcohol than the Chemotion dataset. NIST dataset contains approximately 13% more alcohols than Chemotion dataset. These groups have the highest (relative) difference in the distribution between the Chemotion dataset and the NIST dataset.

**Fig. 2:**
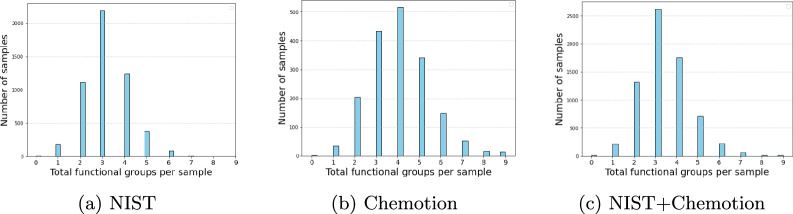
Functional groups present per sample on an average in **a** NIST, **b** Chemotion, and **c** NIST + Chemotion subset

Figure 2 illustrates the distribution of functional groups per sample in the datasets. In Fig. 2a, the NIST dataset displays a peak in the number of samples with three functional groups, and a smaller number with 0 and maximum 7 functional groups. The Chemotion distribution in Fig. 2b indicates a prevalence of samples with four functional groups, with some instances reaching up to nine functional groups. Fig. 2c combines the cumulative distribution of NIST and Chemotion datasets. Notably, due to Chemotion being approximately 33% of the size of the NIST dataset, the cumulative distribution resembles the shape of the larger NIST dataset. This analysis aims to lay the groundwork for later sections, where we investigate the error rates associated with different numbers of functional groups in the samples.

**Fig. 3:**
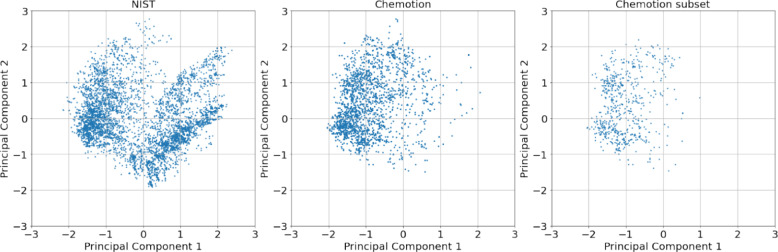
The figure presents a scatter plot depicting the projection of datasets onto the principal components derived from the NIST dataset. Both the Chemotion and Chemotion subset datasets are included in this analysis, allowing for an examination of similarities or differences between Chemotion datasets and the NIST dataset

For the experiments in this study, we used the three datasets (1) NIST, (2) NIST + Chemotion, and (3) NIST + Chemotion subset. A subset of the Chemotion dataset was selected by permutations of molecules containing a particular functional group. This method of creating subsets was applied to improve the performance of the model trained on the combined NIST and Chemotion subset. The Chemotion dataset was sliced based on samples featuring either a singular functional group or a combination of a maximum of two functional groups. The specific Chemotion subset examined in this study resulted from a subset with molecules containing nitriles or alkyl halides. This subset contains 571 samples. It is important to note that this designation does not imply exclusive compositions of nitriles and alkyl halides in the samples but rather signifies the presence of at least one of these functional groups alongside others. In simple terms, it means that the distribution of the sliced dataset facilitates better generalization, and not the presence of nitriles and alkyl halides in the samples. We show this in the results section. To further analyse the similarities between the NIST and Chemotion datasets, we employ a principal component analysis (PCA) technique to the Morgan fingerprints extracted from the NIST dataset. We then utilize this fitted PCA model to transform and visualize the Morgan fingerprints of the Chemotion dataset. This process involves capturing the underlying structure and patterns inherent in the NIST dataset through PCA transformation. By extracting principal components that encapsulate the maximum variance within the NIST data, the PCA model effectively reduces the dimensionality of the dataset while retaining crucial information. This enables a comparative analysis between the datasets within a lower-dimensional space, offering insights into their similarities and differences. Figure 3 shows the scatter plot of the datasets projected onto the principal components of the NIST dataset. It can be observed from the scatter plot of the Chemotion dataset that data points are projected onto a similar space as that of NIST. The density of the Chemotion dataset is close to one of the clusters of the NIST dataset with very few data points in another cluster. This observation suggests that there are underlying similarities or relationships between the datasets, indicating shared characteristics captured by the principal components. Chemotion subset’s density is concentrated around the same space as Chemotion’s density.

## Methods

### Data preprocessing

**Fig. 4:**
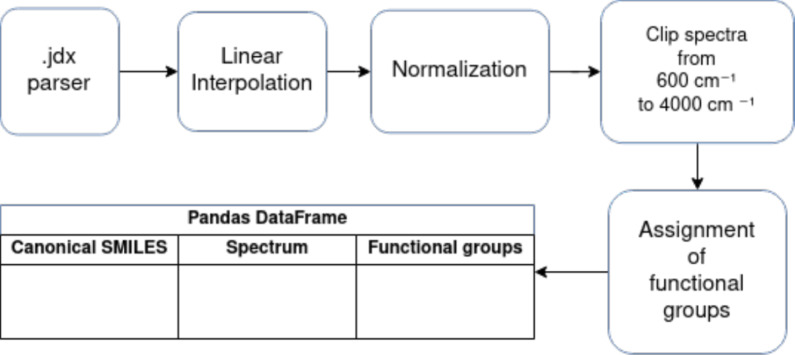
Data preprocessing pipeline

Figure 4 shows the data preprocessing pipeline implemented for our model. A parser is used to parse .jdx files of individual molecules into arrays. Linear interpolation is then carried out to interpolate missing values of the spectrum. Linear interpolation estimates a missing value based on its immediate neighbors, ensuring that the local trend or slope of the spectrum is maintained. The spectrum intensity is then normalized to be between 0 and 1. Since there are different data sources involved, there are different ranges of available wavenumbers. Therefore, we chose to standardize the spectra to a range of 600 $$\textrm{cm}^{-1}$$ to 4000 $$\textrm{cm}^{-1}$$. To find labels for the functional groups present in the molecules, we use the identification method and SMARTS strings definition used by Fine et al. [9] in their work. Instead of InChI strings, we used SMILES codes of the molecules as the input for the functional group identification method. In addition, modifying the SMARTS string for ethers [6] proved essential for clearly distinguishing between ether and ester functional groups. Using the current SMARTS definition for ethers [OD2]([#6])[#6] without further checks or requirements, esters are also identified and incorrectly labeled as ethers. To address this issue, we implemented a corrected version of the SMARTS string: [OD2]([#6;!$(C=O)])([#6;!$(C=O)]). Here, ;!$(C=O) excludes any pattern where carbon is directly bonded to oxygen in a carbonyl group (C=O), ensuring that the ether carbon atom is not part of a carbonyl group.

Functional groups are assigned with binary indicators, 1 indicates the presence, and 0 indicates the absence of the functional groups. Then, the information of the molecules, i.e. canonical SMILES code, IR spectrum array, and functional group labels are stored in a dataframe. Additionally, for our in-house Chemotion dataset of 4175 samples, 441 spectra with high baselines and high background noise were excluded. This was done manually based on visualization and comparing the spectra. According to Chemotion’s data storage protocol, each molecule in this dataset may have multiple samples, leading to multiple spectra available per chemical structure (molecule). Usually, scientists upload additional spectra in those cases where a better quality of the spectrum was obtained. Therefore, our approach involves selecting the most recent measured spectra (assumed to be the spectra with the highest quality) from these multiple samples. Following these steps, we end up with a set of 1763 samples. We did not apply any data cleaning procedure to the commercial NIST dataset.

### Neural network

The neural network optimization process started with a three-layer fully connected architecture, as described in detail in Ref. Fine et al. [9]. Subsequently, a comprehensive hyperparameter optimization was conducted for both the conventional fully connected neural network and the split network (see SI). For the former, the search included the following parameters: Number of hidden layers, hidden layer size, learning rate, batch size, and the number of epochs. In contrast, the split network’s hyperparameter search incorporated an additional parameter - The wavenumber used to partition the spectra.

As an IR spectrum has distinct information contained in the fingerprint region and functional group region, using a single neural network leads to learned latent variables which are a function of both regions of IR spectra. We enforce an inductive bias by learning two separate representations of the two regions before joining them to predict joint results. Thus our network can be formalized as:

1 $$\begin{aligned} y = f_{\text {joint}, \theta _{\text {joint}}} (h_1, h_2) \end{aligned}$$

with learned features $h_1 = f_{\text {FP}, \theta _{\text {FP}}}(x_{\text {FP}})$ and $h_2 = f_{\text {FG}, \theta _{\text {FG}}}(x_{\text {FG}})$, where $x_{\text {FP}}$ represents the fingerprint part of the IR spectrum, $x_{\text {FG}}$ represents the functional group part of the IR spectrum, and $\theta _{\text {joint}}$, $\theta _{\text {FP}}$, $\theta _{\text {FG}}$ being the weights of the three neural networks $f_{\text {joint}}$, $f_{\text {FP}}$, and $f_{\text {FG}}$ in the overall architecture.

**Fig. 5:**
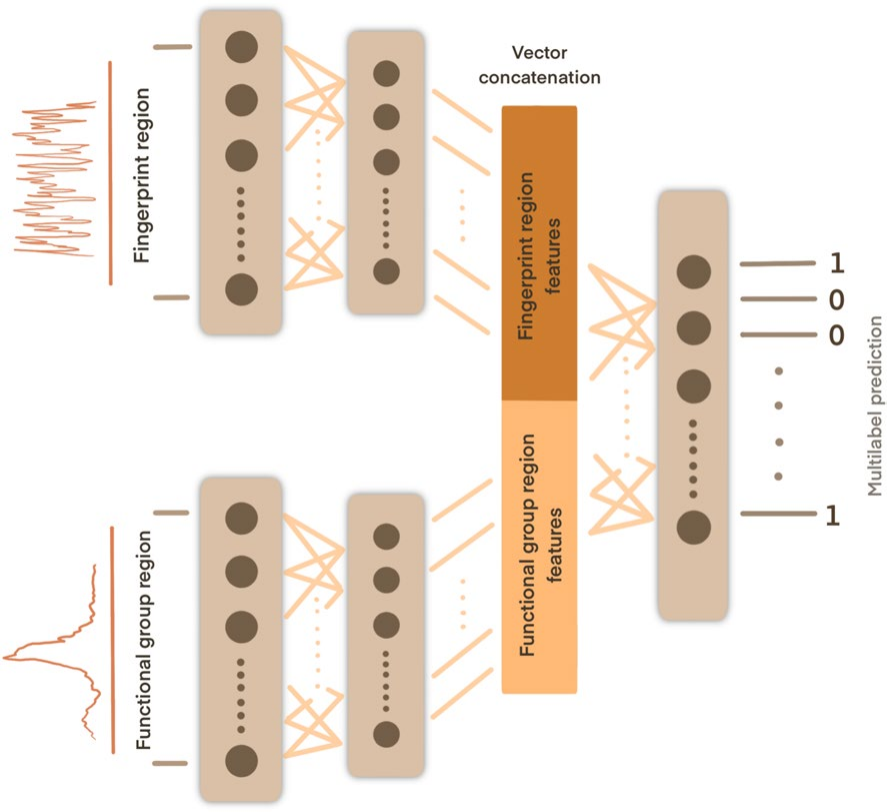
Our network architecture consists of two input heads. Each input head consists of one input layer and one hidden layer. The learned feature vectors are concatenated and passed to another fully connected layer. The output of the last layer is a multilabel prediction, 1 if the respective functional group is present and 0 if the functional group is not present

Our split network architecture is based on the idea of learning two separate representations of distinct parts of the IR spectra, the noisy part and the cleaner part of the spectra. These regions are usually known as the ’fingerprint’ region and the ’functional group’ region, respectively. Therefore, the model has two separate input processing units which both have multiple densely connected layers. The features learned by these units are then concatenated and further processed by a single joint densely connected neural network to generate the functional group detection output (see Fig. 5). The model that is splitting spectra at 1800 $\textrm{cm}^{-1}$ wavenumber showed the best performance. Therefore we select this model for further experiments. Further details about hyperparameter search can be found in the supplementary information section. Table S1 shows the optimal network architectures chosen from hyperparameter search (Table 1).

**Table 1:** Network architecture

	Layers	Dropout	Batch Normalization
Fingerprint head	Layer 1 size: Input size	0.2	Yes
	Layer 2 size: 256	0.3	Yes
Functional group head	Layer 1 size: Input size	0.2	Yes
	Layer 2 size: 256	0.3	Yes
Concat layer	Layer 1 size: 512	–	No

### Experimental design

We utilize a supervised learning approach in a multi-label classification scenario. The labels are represented as binary vectors, where a value of 1 indicates the presence of a corresponding label, and 0 indicates its absence. To ensure unbiased performance evaluation, we divided the datasets into $80\%$ training and $20\%$ validation sets. Since the datasets exhibit class imbalance, it is crucial to mitigate any performance bias resulting from the training split. Hence, we employ a K-fold cross-validation protocol with 5 folds. For training the neural network, we utilize the Adam optimizer, which minimizes the binary cross-entropy loss while employing linear learning rate decay. The reported results are the average validation scores obtained across the 5 folds. We trained the models over 50 epochs, depending on the specific experiment. On average, the 5-fold cross-validation process for our split model takes approximately 1 hour on a system with a Nvidia GeForce 1080 Ti GPU and an Intel Xeon CPU.

## Results

We introduce 3 split neural models based on the datasets used for training:

- Own NIST (trained on NIST dataset)
- Own NIST + Chemotion (trained on NIST and Chemotion dataset)
- Own NIST + Chemotion sub (trained on NIST and a subset of Chemotion dataset)

**Fig. 6:**
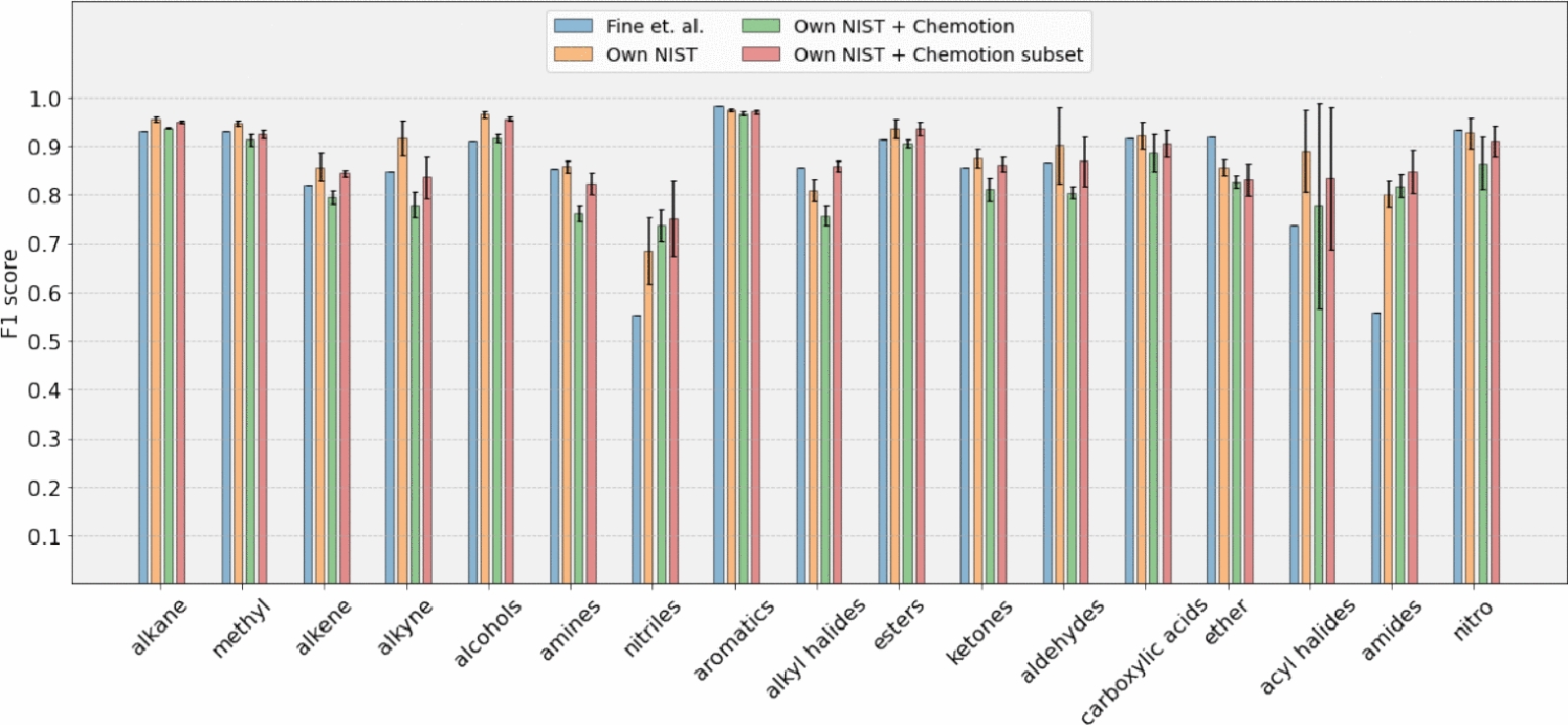
Figure shows the mean F1 scores of the validation set for each functional group. The bars are grouped by different datasets and models

To get an evaluation of how much our work differs from the current state of the art, we compare the F1 scores of our models with the baseline presented in Fine et al. [9]. The F1 score metric we use in our work is same as ’molecular F1 score’ used by Fine et al. [9]. Table 2 and Fig. 6 show the F1 score comparison for 17 functional groups. Although our models demonstrate better average scores compared to the baseline model, the overlapping error bars indicate that the difference may not be statistically significant. Further analysis with a larger sample size or additional metrics may be necessary to confirm the robustness of these results. Our method shows substantial improvement in average scores for amides and nitriles (groups for which the baseline method has the lowest average scores) for all models. A high standard deviation is observed between folds for acyl halides due to the number of samples being as low as 26 in the dataset. Fine et al. [9]’s dataset contained 7393 samples while NIST dataset contained 5228 samples.

**Fig. 7:**
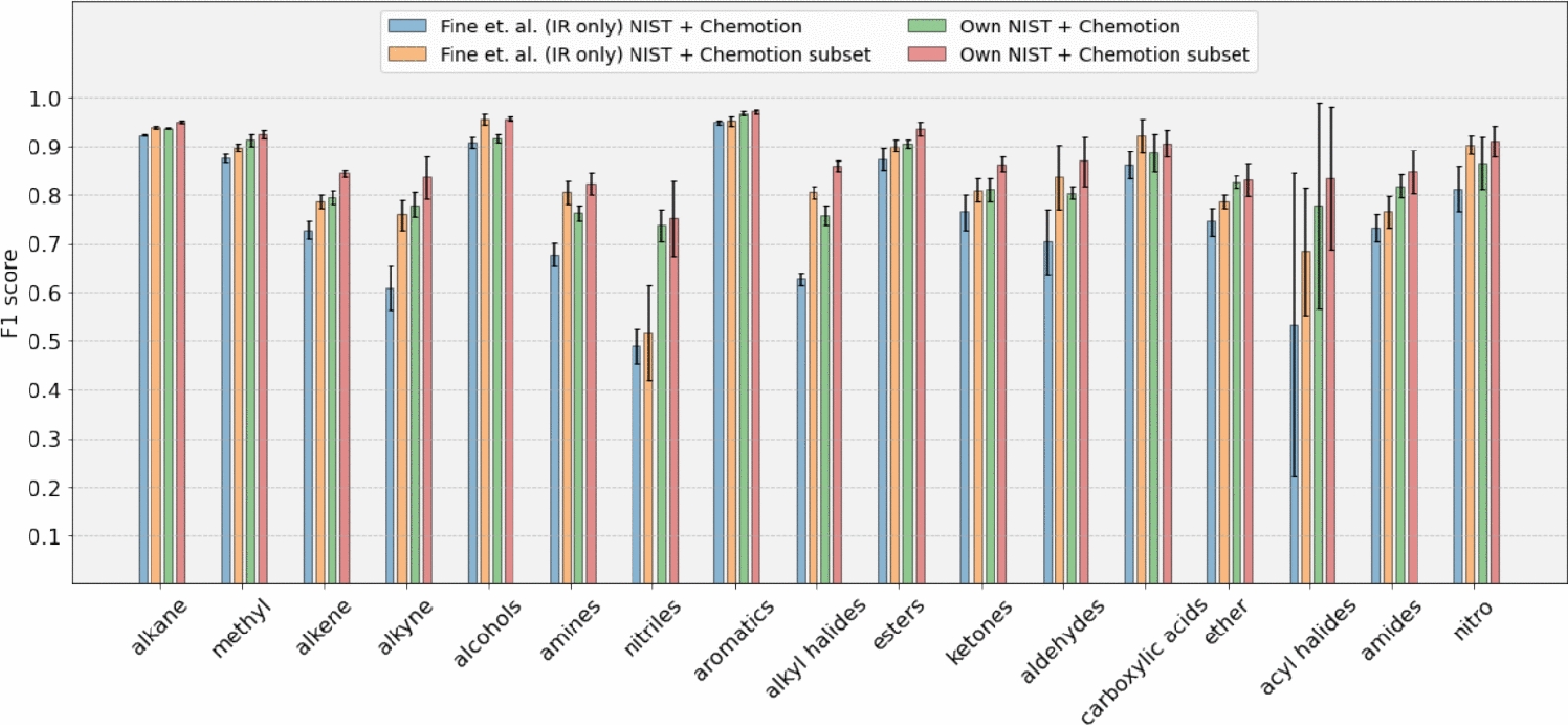
Figure shows the mean F1 scores of the validation set for each functional group. The methods compared in this plot are our model ’Own NIST + Chemotion’ and the retraining of Fine et al.’s “IR spectra only” model with our NIST + Chemotion dataset

Investigating the effect of adding real-world data to the training datasets (obtaining “Own NIST + Chemotion” model) indicates that the performance is slightly reduced when incorporating real-world data, despite the presence of outliers. The mean absolute difference between the “Own NIST” model and the “Own NIST + Chemotion” model is 0.056 F1 score units, while between “Own NIST” and “Own NIST + Chemotion sub” is 0.029 F1 score units. Adding a subset of the Chemotion dataset reduces the error bar of nitriles which signifies a decrease in the variability or uncertainty, indicating an improved level of precision and confidence in the validation set results. While examining the relationship between the number of samples and classification performance, no discernible positive correlation was identified. For instance, a comparison between Figs. 1 and 6 reveals that certain groups, such as aldehydes and alkynes, constitute only 1% and 3% of the molecules, respectively, yet exhibit a high F1 score. This lack of correlation aligns with findings from prior studies Fine et al. [9], Jung et al. [14]. Table 3 shows scores for retraining of Fine et al.’s method with IR data as the only input and the ’NIST + Chemotion’ dataset as training data, evaluated with a 5-fold cross-validation protocol. Ours and Fine’s methods both show improved classification performance for the NIST + Chemotion subset as shown in Fig. 7. Our method has an average score of 0.839 for the NIST + Chemotion dataset and 0.877 for the NIST + Chemotion subset dataset. Compared to that, Fine’s method achieved an average score of 0.753 for the NIST + Chemotion dataset and 0.825 for the NIST + Chemotion subset dataset. Our method achieves F1 scores of 0.73 and 0.75 for nitriles in the NIST + Chemotion and NIST + Chemotion subset datasets, respectively. In comparison, Fine et al.’s method shows F1 scores of 0.48 and 0.51 for the same datasets.

**Table 2:** F1 score comparison with the data of Fig. 6

Functional group	Fine et al. [9]	Own NIST	Own NIST	Own NIST
			+Chemotion	+Chemotion sub
Alkane	0.9323	0.9562$\pm 0.01$	0.9377$\pm 0.0$	0.9515$\pm 0.0$
Methyl	0.931	0.9475$\pm 0.0$	0.9139$\pm 0.01$	0.925$\pm 0.01$
Alkene	0.8196	0.8579$\pm 0.03$	0.7965$\pm 0.01$	0.8443$\pm 0.01$
Alkyne	0.8481	0.9176$\pm 0.04$	0.7803$\pm 0.03$	0.837$\pm 0.04$
Alcohols	0.911	0.9656$\pm 0.01$	0.9173$\pm 0.01$	0.9564$\pm 0.0$
Amines	0.853	0.8592$\pm 0.01$	0.7639$\pm 0.02$	0.8225$\pm 0.02$
Nitriles	0.5533	0.6859$\pm 0.07$	0.7387$\pm 0.03$	0.7524$\pm 0.08$
Aromatics	0.9826	0.9759$\pm 0.0$	0.9682$\pm 0.0$	0.972$\pm 0.0$
Alkyl halides	0.8556	0.8105$\pm 0.02$	0.7583$\pm 0.02$	0.8594$\pm 0.01$
Esters	0.9138	0.9366$\pm 0.02$	0.9062$\pm 0.01$	0.9362$\pm 0.01$
Ketones	0.8572	0.8758$\pm 0.02$	0.8121$\pm 0.02$	0.8635$\pm 0.01$
Aldehydes	0.8667	0.9023$\pm 0.08$	0.805$\pm 0.01$	0.8697$\pm 0.05$
Carboxylic acids	0.9171	0.9228$\pm 0.03$	0.888$\pm 0.04$	0.9057$\pm 0.03$
Ether	0.9191	0.8565$\pm 0.02$	0.8268$\pm 0.01$	0.8318$\pm 0.03$
Acyl halides	0.7368	0.8907$\pm 0.08$	0.7789$\pm 0.21$	0.8339$\pm 0.15$
Amides	0.5578	0.8019$\pm 0.03$	0.8188$\pm 0.02$	0.8479$\pm 0.04$
Nitro	0.9334	0.9281$\pm 0.03$	0.8659$\pm 0.05$	0.9108$\pm 0.03$

**Table 3:** Reproduction of Fine. et. al.’s method (IR only) on NIST + Chemotion dataset

Functional group	Fine et al. [9]	Fine et al. [9]	Own NIST	Own NIST
	NIST+Chemotion	NIST+Chemotion sub	+Chemotion	+Chemotion sub
Alkane	0.9236$\pm 0.0$	0.9388$\pm 0.0$	0.9377$\pm 0.0$	0.9515$\pm 0.0$
Methyl	0.8747$\pm 0.01$	0.8981$\pm 0.01$	0.9139$\pm 0.01$	0.925$\pm 0.01$
Alkene	0.728$\pm 0.02$	0.7878$\pm 0.01$	0.7965$\pm 0.01$	0.8443$\pm 0.01$
Alkyne	0.6096$\pm 0.05$	0.7594$\pm 0.03$	0.7803$\pm 0.03$	0.837$\pm 0.04$
Alcohols	0.91$\pm 0.01$	0.9559$\pm 0.01$	0.9173$\pm 0.01$	0.9564$\pm 0.0$
Amines	0.6785$\pm 0.02$	0.8066$\pm 0.02$	0.7639$\pm 0.02$	0.8225$\pm 0.02$
Nitriles	0.4885$\pm 0.04$	0.5172$\pm 0.1$	0.7387$\pm 0.03$	0.7524$\pm 0.08$
Aromatics	0.9491$\pm 0.0$	0.9519$\pm 0.01$	0.9682$\pm 0.0$	0.972$\pm 0.0$
Alkyl halides	0.6262$\pm 0.01$	0.8061$\pm 0.01$	0.7583$\pm 0.02$	0.8594$\pm 0.01$
Esters	0.8743$\pm 0.02$	0.9018$\pm 0.01$	0.9062$\pm 0.01$	0.9362$\pm 0.01$
Ketones	0.7643$\pm 0.04$	0.811$\pm 0.02$	0.8121$\pm 0.02$	0.8635$\pm 0.01$
Aldehydes	0.7036$\pm 0.07$	0.8371$\pm 0.07$	0.805$\pm 0.01$	0.8697$\pm 0.05$
Carboxylic acids	0.8613$\pm 0.03$	0.9216$\pm 0.03$	0.888$\pm 0.04$	0.9057$\pm 0.03$
Ether	0.7452$\pm 0.03$	0.7881$\pm 0.01$	0.8268$\pm 0.01$	0.8318$\pm 0.03$
Acyl halides	0.5335$\pm 0.31$	0.6842$\pm 0.13$	0.7789$\pm 0.21$	0.8339$\pm 0.15$
Amides	0.7324$\pm 0.03$	0.7654$\pm 0.03$	0.8188$\pm 0.02$	0.8479$\pm 0.04$
Nitro	0.8126$\pm 0.05$	0.9031$\pm 0.02$	0.8659$\pm 0.05$	0.9108$\pm 0.03$

**Fig. 8:**
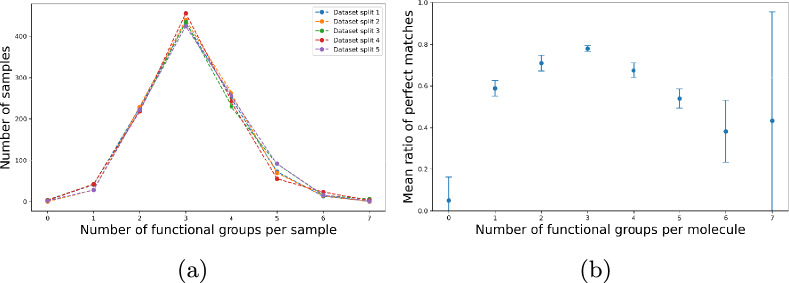
Illustration of the perfect match results for the validation sets across five iterations of a five-fold cross-validation for our ’Own NIST’ model. **a** Count of molecules grouped by the number of functional groups present in them; **b** Mean perfect match ratio of a number of perfect match molecules to the number of total molecules grouped by the number of functional groups present

As there are 0 to 7 functional groups present per sample in the NIST dataset, we explore the classification performance of our model based on the number of functional groups present in the molecules. We extend our model evaluation to assess its perfect match performance, defined as accurately predicting both the present and absent functional groups in a molecule. Fig. 8 illustrates the perfect match performance of our ’Own NIST’ model. Across each validation set in every fold, the model achieves up to a 70% accuracy in predicting molecules with a perfect match. Fig. 8a presents the total number of molecules in each fold, along with the corresponding counts of functional groups. Fig. 8b highlights the mean ratio of the number of perfect matches to the total number of functional groups grouped by the number of functional groups present in the molecule. The perfect match ratio is greater than 0.5 for molecules with at least one functional group and less than 6 functional groups. A very low number of perfect matches are observed for molecules with less than 1 and more than 6 functional groups. This trend correlates to the data distribution as depicted in Fig. 1. As the dataset contains a higher number of samples with 1 to 5 functional groups, the model is better at classifying samples with similar distribution.

**Fig. 9:**
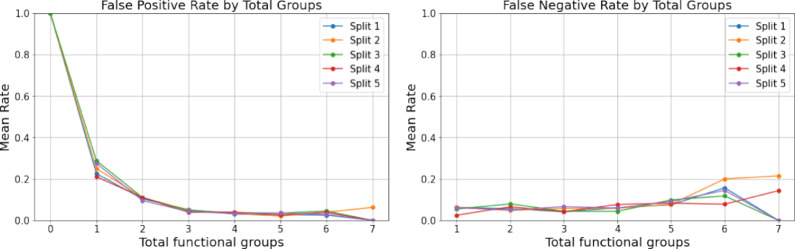
The figure shows the false positive rate and the false negative rate for validation sets of a 5-fold cross-validation of the ’Own NIST’ model. The plot is grouped by the number of functional groups present in molecules depicted by x-axis and the mean false positive and false negative rate depicted by y-axis

Figure 9 illustrates the false positive rate (FPR) and false negative rates (FNR) for the validation sets of a 5-fold cross-validation of the ’Own NIST’ model, grouped by the number of functional groups present in the molecule. Notably, a high FPR is observed for molecules lacking functional groups, consistent with the data distribution depicted in Fig. 9, where there are very few data points without functional groups. The FPR and FNR curves intersect at the same value for samples containing three functional groups, aligning with the highest sample count in this category as shown in Fig. 9. Overall, the FNR remains below 0.2 while the FPR stays under 0.3 (excluding samples lacking any functional groups), indicating the model’s effectiveness in classification when at least one functional group is present. Additionally, Fig. 9 highlights a low number of samples containing seven to eight functional groups; however, the model still demonstrates lower FPR and FNR compared to samples lacking functional groups, despite a similar distribution. These observations suggest the model’s robustness in handling different scenarios and its ability to maintain low error rates across varying compositions of functional groups.

**Fig. 10:**
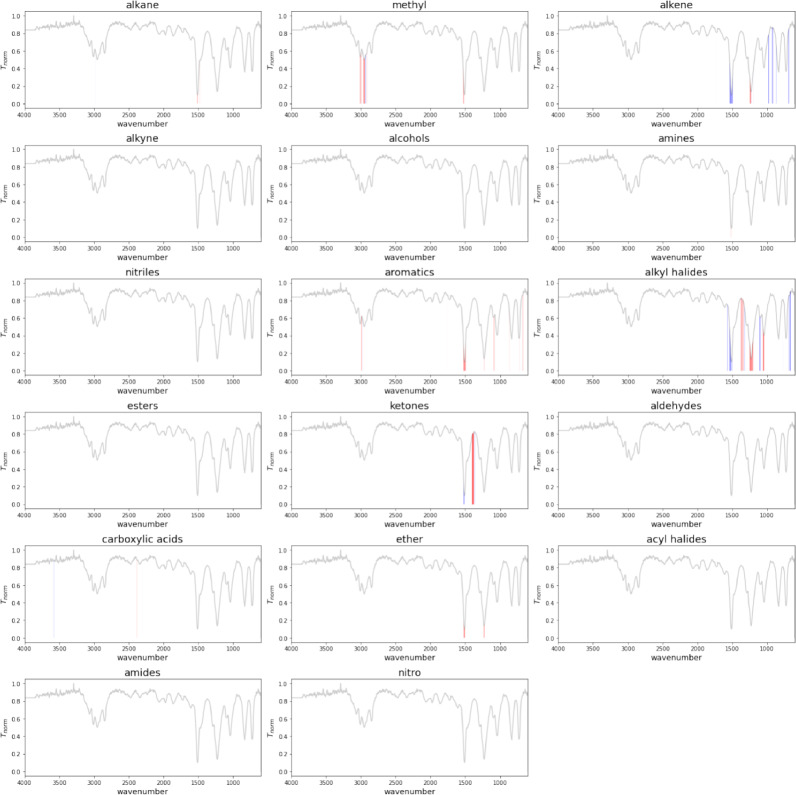
Figure shows SHAP analysis plots for predictions for the input spectrum of the molecule 4-Fluoroanisole COc1ccc(F)cc1 for ’Own NIST’ model. Each plot provides SHAP values for every individual class. Blue regions of the spectrum are influencing the prediction of the model toward predicting the absence of a functional group, while red regions indicates the presence of a given functional group

Figure 10 shows the ’Own NIST’ model’s decision for predicting functional groups from the molecule 4-Fluoroanisole (COc1ccc(F)cc1) with a SHAP (SHapley Additive exPlanations) analysis. The SHAP analysis is a powerful tool in machine learning interpretability, providing insights into individual feature contributions to model predictions [16]. It quantifies the impact of each feature on model output, thereby being beneficial for understanding the decisions of complex models. The molecule contains aromatics, alkyl halides, methyl, and ether groups as ground truths from the dataset. Our model predicts all the labels accurately with a perfect match. Figure 10 shows regions that influence the decision of the model toward predicting the presence of a given functional group (red) and regions which influence the decision of the model towards predicting the absence of the group (blue).

Characteristic IR absorption peaks [4] for functional groups in 4-Fluoroanisole:

Methyl Group (CH$_3$):

- **C-H Stretching**: Around 2970-2860 cm$^{-1}$.
- **C-H Bending**: Around 1470-1370 cm$^{-1}$.

Aromatics:

- **C-H Stretching**: Around 3130-3070 cm$^{-1}$.
- **C=C Stretching**: Around 1615-1580 cm$^{-1}$ and 1510-1450 cm$^{-1}$.
- **C-H Bending**: Typically in the region 900-670 cm$^{-1}$.

Alkyl Halides (Fluorine attached to the benzene ring):

- **C-F Stretching**: Around 1000-1400 cm$^{-1}$ (typically strong and sharp).

Ether Group (C-O-C):

- **C-O Stretching**: Around 1300-1000 cm$^{-1}$ (strong).

As shown in Fig. 10, characteristic signals for the above groups are highlighted in red. Our model utilizes regions of the IR spectrum that align with established chemical principles to identify present functional groups. Influences for groups like alkane, alkene and ketones are also observed in the analysis, but these groups are accurately predicted to be absent in the sample. We present more examples of SHAP analyses in the SI section.

### Holdout testing

**Fig. 11:**
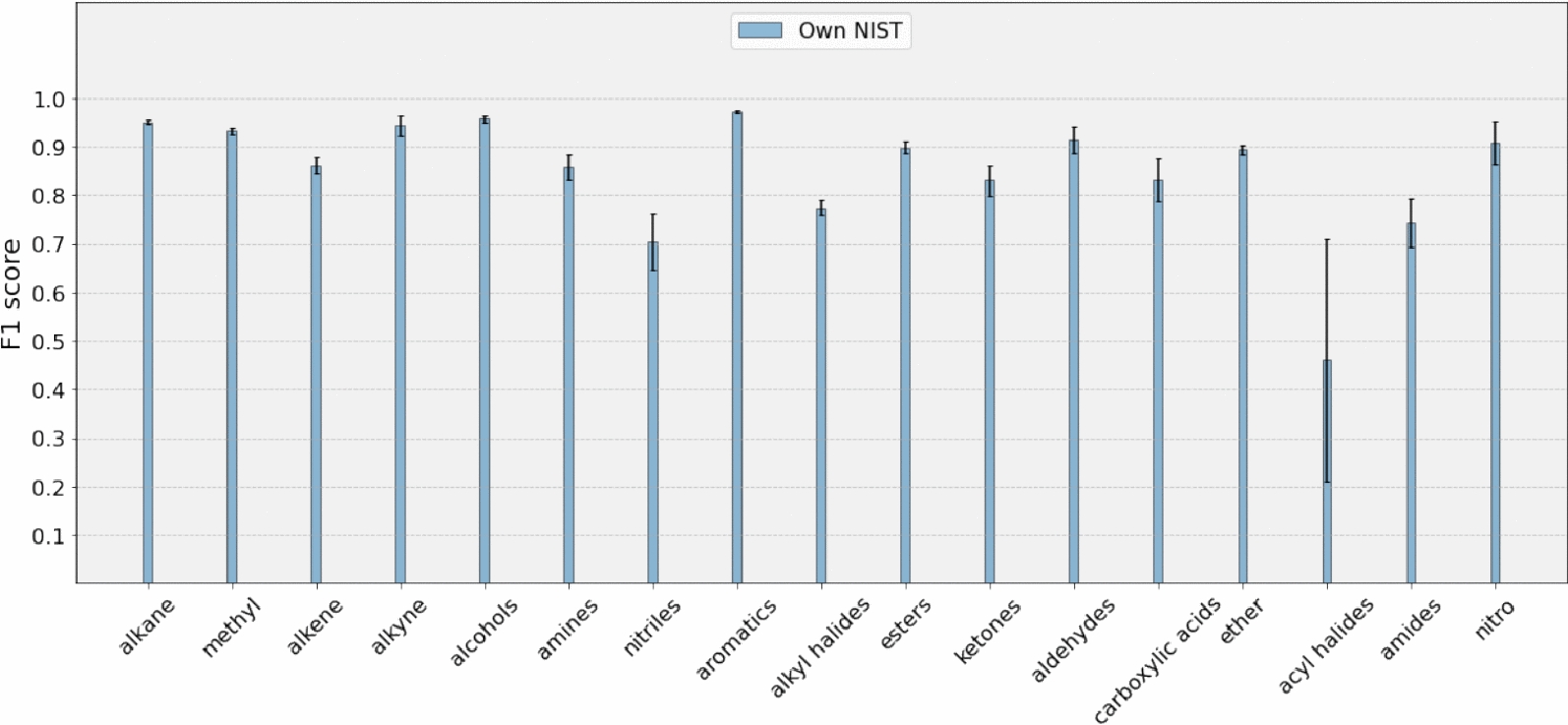
The figure displays the mean F1 scores of our split model across five models from a 5-fold cross-validation, evaluated for each functional group. These scores were calculated using a holdout test set

Holdout testing provides an assessment of model performance on unseen data. We carried out holdout testing for our split network by dividing the dataset into three distinct subsets using a 70-20-10 ratio. We allocated 70% of the data for model training, 20% for validation to fine-tune the hyperparameters and monitor overfitting, and reserved the remaining 10% as a set of tests for final evaluation. We performed 5-fold cross-validation on the training-validation split to evaluate the model’s performance and the hyperparameter tuning. After choosing the best hyperparameter settings we evaluated 5 models from 5 folds on the test set. Fig. 11 shows the mean F1 scores and standard deviation on the test set for 5 models from 5-fold cross validation. Our model’s performance is consistent across all functional groups. Acyl halides have the lowest F1 score of 0.461, which is likely due to the presence of only three acyl halide samples in the test set. Overall, the representation of this group is low in our dataset as shown in Fig. 1. Our network shows consistent performance for nitriles and amides with F1 scores of 0.70 and 0.74 respectively. Further information for hyperparameter search and training testing loss curves are mentioned in the SI section.

## Conclusion

In this study, we present a method to predict the presence of functional groups in chemical compounds based on the measured IR spectroscopic data. Our method uses IR data as the only type of measured input which makes it better and broader usable due to the independence of other data types. Our method ’Own NIST’ uses a deep learning model that performs better than previous work that used a combination of IR spectra and mass spectra inputs. At the same time, our model significantly improves the classification accuracy of nitriles and amides groups. Our method yields predictions that perfectly match 70% of the molecules in the validation set. Besides the provision of the model itself, we demonstrated the integration of open access data [15] available from a research data repository. We were able to show that, despite the fact that the open-access data is in-homogeneous, comparable results could be obtained. Unfortunately, there is a lack of openly available datasets for IR analysis which hinders the further development of data-driven methods. We take a step in this direction by publishing our in-house dataset Chemotion, which contains real-world data produced in different labs, therefore establishing a start for a valuable benchmark dataset for machine learning research. Future work will be directed toward the creation of curated datasets to contain molecular diversity which facilitates better generalizations. As our model is feasible to be integrated with research software, we intend to make our developments directly available in ELNs, fostering a direct use by bench scientists.

## Supplementary Information

Supplementary material 1.

## Data Availability

Code: Github repository , Zenodo [23]. The repository contains the source code and documentation necessary for reproducing the results and further development. Dataset: Radar4Chem Chemotion repository IR Dataset [15]. The dataset contains jcamp files for each sample in Chemotion repository [3].
