# Supplementary material for: Infrared spectrum analysis of organic molecules with neural networks using standard reference data sets in combination with real-world data
Source: J Cheminform. 2025 Feb 26;17:24. doi: 10.1186/s13321-025-00960-2 (PMC11863843; doi:10.1186/s13321-025-00960-2)
Supplement: Supplementary file 1 — Supplementary material 1. [file 13321_2025_960_MOESM1_ESM.pdf]

Supplementary Information

1 Hyperparameter search

To identify the optimal hyperparameter configuration for the neural network, we conducted a hyperparameter search, a crucial step in fine-tuning neural networks. This process involves adjusting parameters such as learning rate, batch size, and regularization to achieve the best model performance. Through iterative exploration of these parameters, we aim to achieve the optimal generalization, ultimately improving the model's predictive capabilities. In this study, we employed grid search for hyperparameter optimization.

Selecting the most suitable model is a challenging task due to the prediction of multiple functional groups. Consequently, for a given model, some groups may exhibit the highest scores, while others may have the lowest scores. In these figures, we present all scores of the model that achieved the maximum score for at least one functional group. Additionally, the figures display the scores of the model with the highest mean score across all functional groups.

The initial neural network design was carried out with a fully connected network. The results for a fully connected network are shown in Figure S1. As there are multiple functional groups involved, different models achieve the best scores for different functional groups. In Figures S1 and S2, we display each model with the best scores

Table S1: Hyper parameter search settings

Hyperparameters	Fully Connected Network settings	Split Network settings
Hidden layer size	128, 256	128, 256
Number of hidden layers	1, 2	1, 2
Dropout rate	0.2, 0.3	0.2, 0.3
Learning rate	0.001, 0.01	0.001, 0.01
Batch size	32, 64	32, 64
Epochs	50, 100	50, 100
Split wave number	-	1600, 1800, 2100

for every functional group along with the model that has the best scores in terms of mean scores of all functional groups. Even though the fully connected model performs well enough for most of the functional groups, it underperforms for the nitrile group. As we aim to produce a method that has good classification performance for all functional groups, we create a split network and perform a hyperparameter search for a split network as well. Figure S2 shows hyperparameter search results for a split network. We select the model with the best mean scores for all functional groups. The following hyperparameters produce the best results:

- Split spectra at: 1800 cm^{-1}
- Hidden layer size: 256
- Batch size: 32
- Number of hidden layers: 2
- Dropout input layer: 0.2
- Dropout hidden layer: 0.3
- Concat layer size: 512
- Learning rate: 0.01
- Epochs: 50

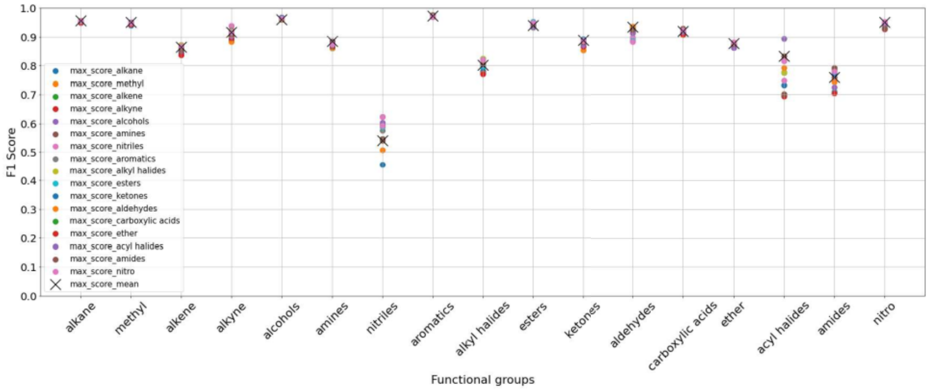

Fig. S1: The plot showcases hyperparameter search results for a fully connected network. Models with maximum score for each functional group and models with maximum mean score for all functional groups are displayed.

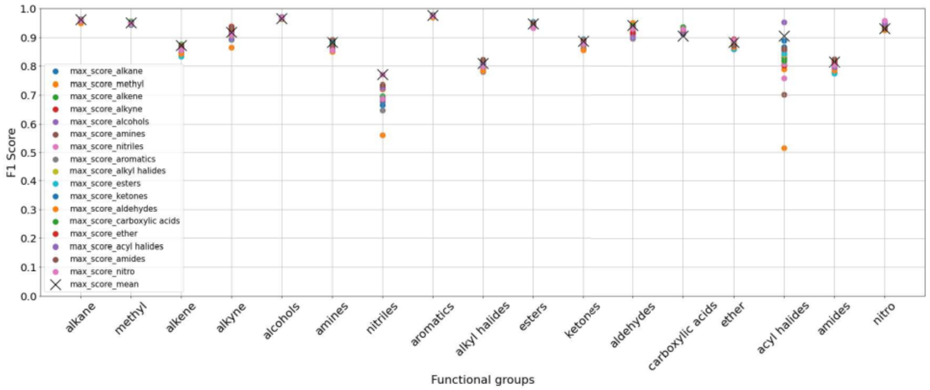

Fig. S2: The plot showcases hyperparameter search results for our split network. Models with maximum score for each functional group and models with maximum mean score for all functional groups are displayed.

2 Holdout testing

Figure S3 shows the results for holdout testing to choose the best-performing model for hyperparameter search for a split network. It is observed that the model with the best mean scores across all functional groups also demonstrates acceptable performance for each functional group.

Figure S4 shows the training and validation curves for the holdout testing. The training loss and validation show minimal deviation throughout the training process.

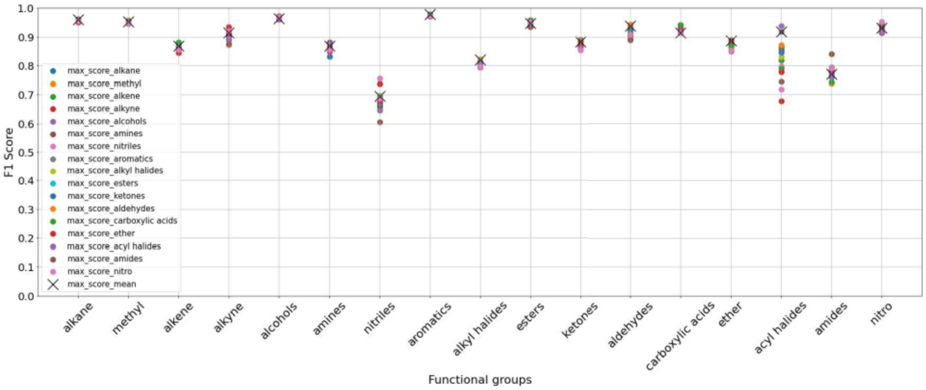

Fig. S3: The plot showcases hyperparameter search results for a split network with holdout testing on the test dataset. Models with the highest score for each functional group and models with the highest mean score across all functional groups are displayed.

Table S2: Reproduction of Fine et. al's method (IR only) on NIST + Chemotion dataset

Functional group	Own NIST NIST
alkane	0.9516±0.0
methyl	0.9326±0.01
alkene	0.8616±0.02
alkyne	0.9436±0.02
alcohols	0.9585±0.01
amines	0.8583±0.03
nitriles	0.705±0.06
aromatics	0.9719±0.0
alkyl halides	0.7745±0.01
esters	0.8993±0.01
ketones	0.8305±0.03
aldehydes	0.9148±0.03
carboxylic acids	0.8325±0.04
ether	0.8945±0.01
acyl halides	0.461±0.25
amides	0.7435±0.05
nitro	0.9086±0.04

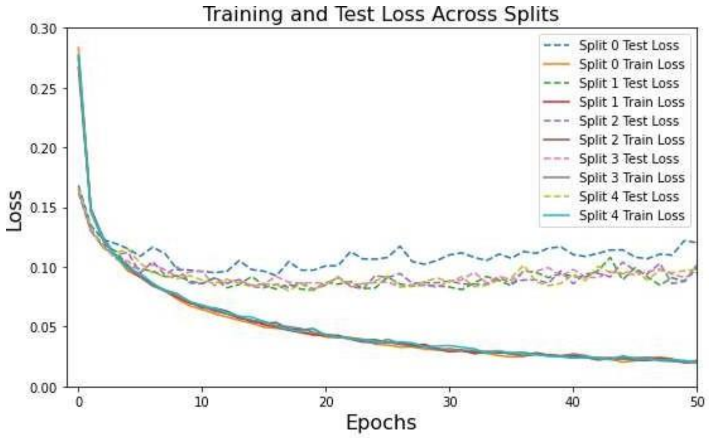

Fig. S4: The figure illustrates the training and test loss for five models generated during 5-fold cross-validation

3 SHAP (SHapley Additive exPlanations) analysis

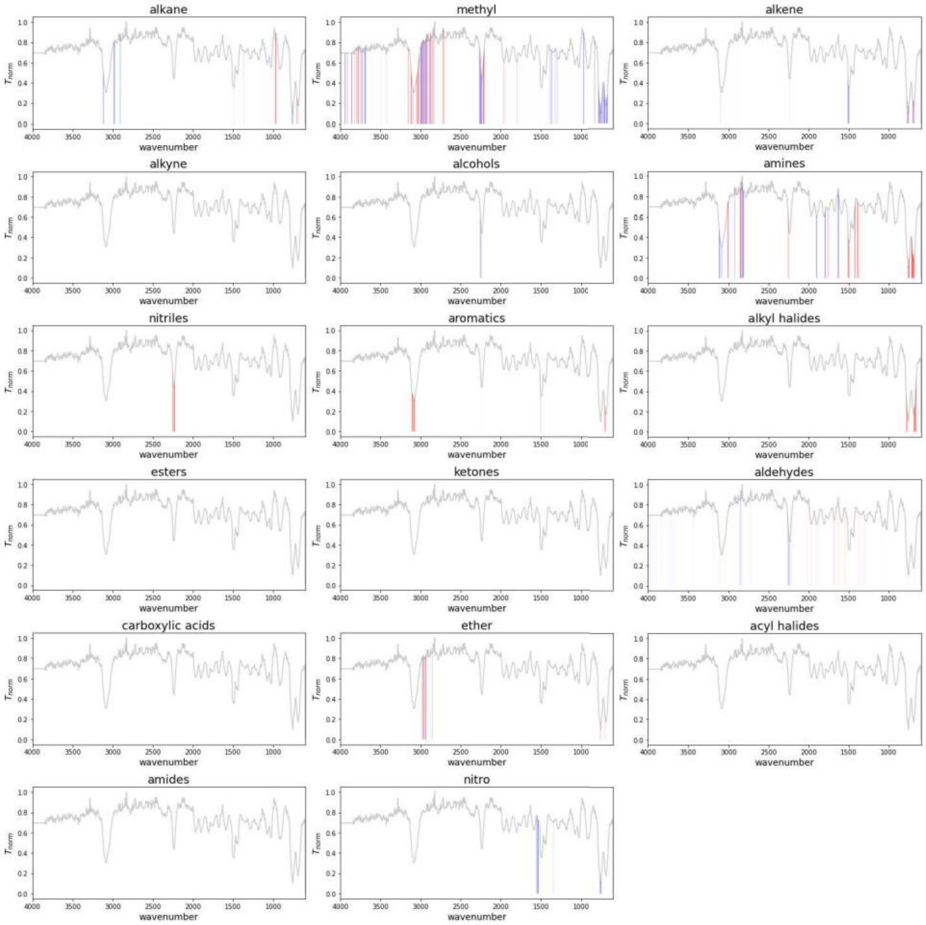

Fig. S5: Figure shows SHAP analysis plots for predictions for the input spectrum of the molecule benzonitrile for 'Own NIST' model. Benzonitrile contains nitriles and aromatics groups. Our model predicts nitriles, aromatics and alkane groups. The figure shows that the model identifies a peak near 2250 cm^{-1} as indicative of both nitriles and alkynes. This region is characteristic of a triple bond stretch. Specifically, alkynes exhibit a C≡C stretch, while nitriles exhibit a C≡N stretch.

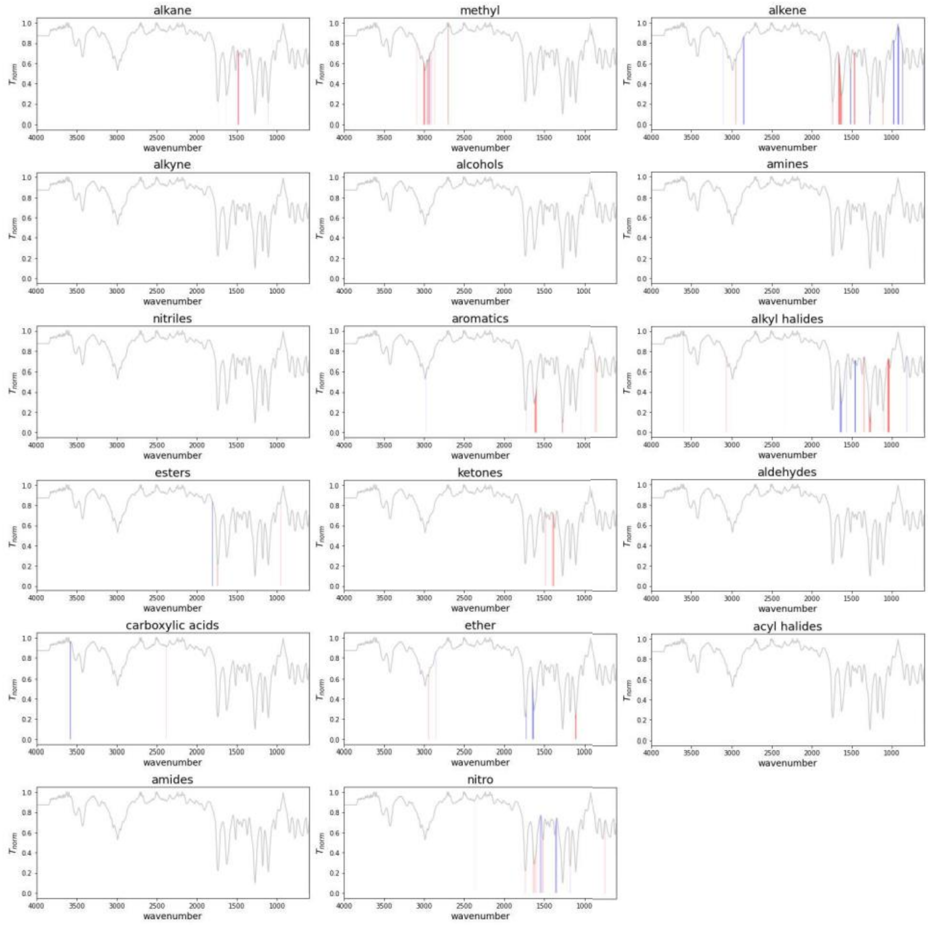

Fig. S6: Figure shows SHAP analysis plots for predictions for the input spectrum of the molecule Ethyl 4-aminobenzoate for 'Own NIST' model. Each plot provides SHAP values for every individual class. Ethyl 4-aminobenzoate contains alkane, methyl, amines, aromatics and ester groups. All these groups are identified by our model with a perfect match. The figure shows that the characteristic ester peak around 1750-1725 cm^{-1} is recognized correctly.

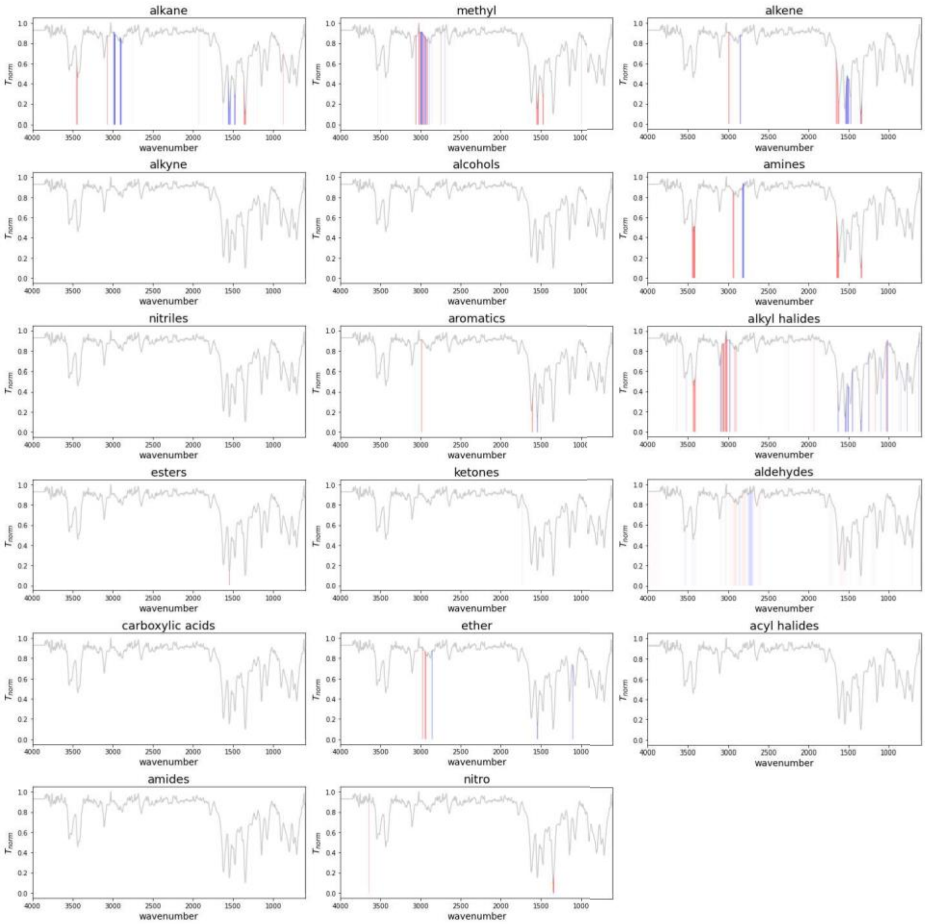

Fig. S7: Figure shows SHAP analysis plots for predictions for the input spectrum of the molecule 2,6-dichloro-4-nitroaniline for 'Own NIST' model. This molecule contains amines, aromatics, alkyl halides and nitro groups. All these groups are identified by our model with a perfect match. The characteristic NH stretch of a aromatic primary amine around 3500 and 3400 cm^{-1} is identified correctly by the model.
